# Durable Responses to Anti-PD1 and Anti-CTLA4 in a Preclinical Model of Melanoma Displaying Key Immunotherapy Response Biomarkers

**DOI:** 10.3390/cancers14194830

**Published:** 2022-10-03

**Authors:** Elena Shklovskaya, Bernadette Pedersen, Ashleigh Stewart, Jack O. G. Simpson, Zizhen Ming, Mal Irvine, Richard A. Scolyer, Georgina V. Long, Helen Rizos

**Affiliations:** 1 Macquarie Medical School, Faculty of Medicine, Human and Health Sciences, Macquarie University, Sydney, NSW 2109, Australia; 2 Melanoma Institute Australia, The University of Sydney, Sydney, NSW 2006, Australia; 3 Faculty of Medicine and Health, The University of Sydney, Sydney, NSW 2006, Australia; 4 Tissue Pathology and Diagnostic Oncology, Royal Prince Alfred Hospital and NSW Health Pathology, Sydney, NSW 2006, Australia; 5 Charles Perkins Centre, The University of Sydney, Sydney, NSW 2006, Australia; 6 Department of Medical Oncology, Northern Sydney Cancer Centre, Royal North Shore Hospital, Sydney, NSW 2065, Australia; 7 Department of Medical Oncology, Mater Hospital, Sydney, NSW 2060, Australia

**Keywords:** immune checkpoint blockade, melanoma, mouse models, antigen presentation, major histocompatibility (MHC) class I, interferon, T lymphocytes, immunological memory

## Abstract

**Simple Summary:**

Immunotherapy has improved the outcomes of patients with advanced melanoma, although many patients will progress while on treatment. Preclinical animal models provide valuable insights into immunotherapy response or resistance and can be used to test novel treatment combinations. The development of animal cancer models rarely involves the systematic analysis and inclusion of predictive biomarkers of immunotherapy response. This study describes a biomarker-driven workflow to generate a transplantable mouse melanoma model responsive to anti-PD1 and anti-CTLA4 immunotherapy. This model recapitulates human immunotherapy-responding tumor phenotypes and provides unique insights into the discrete mechanisms underlying the durability of response to immune checkpoint inhibitors.

**Abstract:**

Immunotherapy has transformed the management of patients with advanced melanoma, with five-year overall survival rates reaching 52% for combination immunotherapies blocking the cytotoxic T-lymphocyte-associated antigen-4 (CTLA4) and programmed cell death-1 (PD1) immune axes. Yet, our understanding of local and systemic determinants of immunotherapy response and resistance is restrained by the paucity of preclinical models, particularly those for anti-PD1 monotherapy. We have therefore generated a novel murine model of melanoma by integrating key immunotherapy response biomarkers into the model development workflow. The resulting YUMM3.3^UVR^c34 (*BrafV600E; Cdkn2a*–/–) model demonstrated high mutation burden and response to interferon (IFN)γ, including induced expression of antigen-presenting molecule MHC-I and the principal PD1 ligand PD-L1, consistent with phenotypes of human melanoma biopsies from patients subsequently responding to anti-PD1 monotherapy. Syngeneic immunosufficient mice bearing YUMM3.3^UVR^c34 tumors demonstrated durable responses to anti-PD1, anti-CTLA4, or combined treatment. Immunotherapy responses were associated with early on-treatment changes in the tumor microenvironment and circulating T-cell subsets, and systemic immunological memory underlying protection from tumor recurrence. Local and systemic immunological landscapes associated with immunotherapy response in the YUMM3.3^UVR^c34 melanoma model recapitulate immunotherapy responses observed in melanoma patients and identify discrete immunological mechanisms underlying the durability of responses to anti-PD1 and anti-CTLA4 treatments.

## 1. Introduction

Immunotherapies targeting the T-cell immune inhibitory receptors, cytotoxic T-lymphocyte-associated antigen-4 (CTLA4) and programmed cell death-1 (PD1), have transformed clinical management of patients with a range of advanced cancers. For advanced melanoma, 5-year overall survival reached 26% for anti-CTLA4 monotherapy, 44% for anti-PD1 monotherapy and 52% for the combined (anti-CTLA4 plus anti-PD1) treatment [1], yet a large proportion of patients exhibit primary resistance. Despite considerable progress towards understanding the molecular mechanisms of immunotherapy resistance, credible preclinical animal models are urgently needed to interrogate response and resistance effectors and test novel treatment combinations and administration schedules. Predictive biomarkers of immunotherapy response and resistance established for human disease, are rarely integrated into or examined in a systematic way in preclinical animal models for cancer immunotherapy.

Important biomarkers predictive of clinical response to immunotherapy in melanoma and other cancers include high tumor mutation burden [2,3], tumor infiltration with CD8 T cells [4,5], tumor expression of the PD1 ligand, PD-L1 [6], immune-related signatures of local T-cell activation and interferon (IFN)γ response [5,7], and early on-treatment changes associated with T-cell activation and IFNγ release [8,9]. In concert with the importance of CD8 T cell-mediated tumor recognition and killing for tumor control, tumor expression of antigen-presenting major histocompatibility class I (MHC-I) molecules [10] and intact IFNγ signaling [11,12] correlate with immunotherapy response, while a deficiency in MHC-I expression [13,14] or IFNγ response [11,12], are associated with immunotherapy resistance.

The Yale University Mouse Melanoma (YUMM) cell series carry genetic alterations relevant to human disease such as the *BrafV600E* activating mutation [15] and have been used extensively in melanoma research, including evaluation of response to targeted therapies [16] and immunotherapies [17,18,19,20]. Exposure of YUMM cells to ultraviolet radiation (UVR) [21,22] or cisplatin [23] increases tumor mutation burden consequently improving tumor immune recognition, and such tumors are either rejected spontaneously [22], controlled with anti-CTLA4, continuous anti-PD1 administration [21], or combined (anti-CTLA4 plus anti-PD1) treatment [23].

We have established a biomarker-driven workflow to generate a transplantable mouse melanoma model responsive to immunotherapy. In this workflow, parental cells were selected based on immunotherapy response biomarkers, UVR mutagenesis was employed to increase mutation burden, and tumor clones were derived based on immune reactivity in vitro. We have established two related models, one yielding progressing tumors that respond to both single agent- and combined immunotherapy, the other resulting in spontaneously regressing tumors. We report immunological determinants distinctly associated with local and systemic immunotherapy response and protective immunological memory for anti-CTLA4 and anti-PD1 monotherapies. Our model faithfully recapitulates human immunotherapy-responding tumor phenotypes, provides unique insights into the discrete mechanisms underlying the durability of response to the two most clinically relevant checkpoint blockers and can be used to explore the molecular mechanisms of immunotherapy resistance.

## 2. Materials and Methods

### 2.1. In Vivo Mouse Studies

All animal studies were performed in accordance with the National Health and Medical Research Council (NHMRC) Australian code for the care and use of animals for scientific purposes and with approval from the Macquarie University Animal Ethics Committee (Animal Research Authority (ARA) 2019-020). Male and female six- to eight-week-old C57BL/6J mice were purchased from the Animal Resources Centre (ARC, Perth, Australia) and allowed to acclimatize for 1–2 weeks prior to use. Unless otherwise specified, sex-matched animals were used in experiments. For tumor grafting, fur on one flank was shaved and tumor cells (0.5 × 10^6^ unless otherwise specified) injected subcutaneously in 50 μL saline. Tumor sizes were measured 2–3 times/week with digital calipers and tumor volume was calculated as (width^2^ × length)/2. Unless otherwise stated, animals were euthanized when tumor volume reached 1000 mm^3^ or when necrosis developed on the skin overlaying the tumor. For immunotherapies, anti-PD1 (clone RMP1-14, Cat BE0146, BioXCell, Lebanon, NH) and anti-CTLA4 (clone 9H10, BioXCell Cat BP0131) were administered by intraperitoneal injection in 200 μL saline. For anti-CTLA4 treatment, three doses (200 μg,100 µg,100 µg) were administered three days apart for a total of three injections; alternatively, 3 doses each of 200 μg were administered three days apart. For anti-PD1 treatment, 300 μg per dose was administered three days apart for a total of five injections; in some experiments, 400 μg or 500 μg per dose was administered three days apart for a total of five injections. Control groups received isotype-matched control antibodies (rat IgG2a for anti-PD1, BioXCell Cat BE0089 and hamster IgG for anti-CTLA4, BioXCell Cat BE0087).

### 2.2. Human Melanoma Studies

This study cohort was previously reported in [13] and included 17 patients treated with anti-PD1 monotherapy (nivolumab or pembrolizumab), with pre-treatment viable melanoma biopsies available for flow cytometry analysis.

### 2.3. Maintenance of Melanoma Cell Lines and Generation of Clones

The *BrafV600E* YUMM and YUMMER cell lines YUMM1.1, YUMM1.7, YUMM2.1, YUMM3.3 [15] YUMMER1.7 [21] and YUMMER.G [22] have previously been described and were kindly provided by M. Bosenberg. YUMMER lines carry multiple somatic mutations due to UVR exposure. Cell lines were cultured in complete medium (RPMI-1640 containing 10% heat-inactivated fetal bovine serum (FBS), 10 mM HEPES, 1% non-essential amino acids, 2 mM L-glutamine (all from Sigma Aldrich, St. Louis, MO, USA), 2 mM L-glutamine and 55 μM 2-mercaptoethanol (Gibco, Thermo Fisher Scientific, Waltham, MA, USA)). Cells were maintained at 37 °C in 5% CO_2_. All cell lines tested negative for mycoplasma (MycoAlert Mycoplasma Detection Kit, Lonza, Basel, Switzerland). For in vivo studies, cells (batch-frozen and cryopreserved after initial expansion on arrival), were thawed and expanded (up to 3 passages) before being injected into animals. For IFNγ treatment, melanoma cells were plated in 6-well plates (10^5^ cells per well). After overnight incubation, medium was replenished, and recombinant murine (rm) IFNγ (Peprotech, Rocky Hill, NJ, USA) was added; the dose was 1ng/mL unless otherwise specified. For vehicle control, 0.1% bovine serum albumin (Sigma-Aldrich, St. Louis, MO, USA) in phosphate-buffered saline (PBS, Gibco, Waltham, MA, USA) was used.

To generate YUMM3.3^UVR^ clones, YUMM3.3 (Y3.3) cells were seeded in 6-well plates at 10^5^ cells/well and allowed to rich 30–40% confluency. Plates were UV-irradiated with 0.15 J/cm^2^ using a UVC source (UVILink CL-508, Uvitec, Cambridge, UK). Surviving clones were expanded, re-seeded as above and irradiated again, for a total of five UV cycles. Following the last UV treatment and recovery, cells were seeded for single-cell cloning. The resulting clones were expanded and cryopreserved. In addition, cells were stimulated with IFNγ or vehicle to assess baseline and IFNγ-induced MHC-I expression by flow cytometry, and cell pellets (T25 at 70–80% confluency) were frozen (−80 °C) for immunoreactivity tests.

### 2.4. T Cell Proliferation Assay

To generate dendritic cells (DCs), bone marrow was flushed from tibiae and femora and 2 × 10^7^ cells (0.5 × 10^6^/mL) cultured in complete medium supplemented with 20 ng/mL recombinant mouse (rm)GM-CSF and 5 ng/mL rmIL-4 (both from Peprotech, Rocky Hill, NJ, USA) for 6 days; cytokines were replenished on days 2 and 4 [24]. On day 6, DCs were collected, washed and seeded in 24-well plates at 10^6^ DCs/well. DCs were pulsed overnight with tumor cell lysates. For cell lysates, frozen cell pellets (2 × 10^6^ cells) of the selected Y3.3^UVR^ clones were lysed by 5 rounds of freezing/thawing. On the next day, DCs were washed and plated in triplicate in flat bottom 96-well plates, at 5000 and 10,000 cells/well. To prepare T cells, pooled lymph nodes were teased through a stainless-steel mesh, washed and labeled with the carboxyfluorescein succinimidyl ester (CFSE) CellTrace (Thermo Fisher Scientific, Waltham, MA, USA) according to the manufacturer’s instructions. CFSE-labeled cells (1 × 10^5^) were added to each well, to obtain a T cell: DC ratio of ratio 1:10 or 1:20. T cell proliferation (CFSE dilution) was assessed by flow cytometry after 6 days of culture.

### 2.5. Cell Proliferation Assay (IncuCyte)

Melanoma cells were seeded into 96 well flat bottom clear plates (1 × 10^3^ cells/well) and after an overnight incubation, medium was replenished and cells were treated with 1 ng/mL, 10 ng/mL or 100 ng/mL rmIFNγ (Peprotech, Rocky Hill, NJ, USA) or vehicle control in triplicates. Cells were analyzed on the IncuCyte ZOOM live cell imaging system (Sartorius, Goettingen, Germany). Four images per well were taken every 4 h using the default software parameters for a 96-well plate (Corning) with a 10× objective. The IncuCyte software was used to calculate mean confluence from four non-overlapping bright phase images for each well.

### 2.6. Mouse Tissue Processing and Flow Cytometry Analysis

Tumors were manually cut with surgical blades and enzymatically dissociated using the mouse tumor dissociation kit and gentleMACS Dissociator (Miltenyi Biotec, Bergisch Gladbach, Germany), as directed by the manufacturer. Sentinel lymph nodes (pooled ipsilateral inguinal and axillary) [25] were teased through a stainless-steel mesh. Tissue samples were cryopreserved in 10% DMSO and slowly thawed for flow cytometry analysis. Ethylenediaminetetraacetic acid (EDTA)–blood obtained via submandibular vein sampling (longitudinal) or cardiac puncture (post-euthanasia), were treated with red cell lysis buffer (Sigma). Processed bloods were cryopreserved in 10% DMSO, and both fresh and thawed blood samples were used for flow cytometry analysis.

Staining for flow cytometry was performed in flow cytometry buffer (saline containing 5% FBS, 10 mM EDTA and 0.05% sodium azide). Cells were initially incubated with Fc block (BD Biosciences) to prevent non-specific staining due to Fc receptor binding. In the second step, samples were incubated for 30 min on ice with fluorescently labeled monoclonal antibodies directed against cell surface markers (reagents are listed in Table S1). Live Dead near-infrared fixable dye (Invitrogen, Thermo Fisher Scientific, Waltham, MA, USA) was used for dead cell exclusion. After fixation and permeabilization with the eBioscience transcription factor buffer set (Thermo Fisher Scientific, Waltham, MA, USA) used as recommended by the manufacturer, cells were incubated with fluorescently labeled monoclonal antibodies directed against intracellular targets. BD LSR Fortessa X20 flow cytometer (BD Biosciences) was used for acquisition, and FlowJo v10 (BD) for data analysis. Relative marker expression was calculated as median fluorescence intensity (MFI) of the antibody-stained sample divided over MFI of unstained control (single markers) or fluorescence minus one (FMO) control (staining with a full antibody panel bar the channel of interest).

### 2.7. Human Tissue Processing and Flow Cytometry Analysis

Tumor biopsies were processed, cryopreserved as viable dissociates and analyzed as previously reported [13]. Key antibodies are listed in Table S1. For further details including the gating strategy, see [13].

### 2.8. DNA Extraction and Whole Exome Sequencing (WES)

DNA was extracted from Y3.3, Y3.3^UVR^c2 and Y3.3^UVR^c34 cell lines as well as wild-type C57BL/6J mouse blood using QIAamp DNA Mini Kit (Qiagen, Hilden, Germany). Exonic DNA was enriched using the Agilent SureSelect Mouse Target enrichment kit and sequenced on an Illumina HiSeq sequencer by Macrogen Oceania (Seoul, South Korea). Reads were mapped to the mm10 reference (original GRCm38) using BWA version 0.7.10. Duplicate reads were marked with Picard 1.118 and applied GATK indel realignment and base quality recalibration. Single nucleotide variants (SNVs) and small insertion/deletions (INDELS) were detected by GATK3.v4, and the variants were annotated using the mm10 dbSNP142 and Indel databases using SnpEff_v4.1. To generate a list of high-quality variants we removed germline (derived from C57BL/6J blood sequence) and dbSNP142 variants. Low coverage variants (read depth < 30 and allele depth < 10) were also removed, and missense mutations were reported.

### 2.9. Statistical Analyses

Statistical analyses were performed with GraphPad Prism version 9.2 (GraphPad Software, La Jolla, CA, USA); a *p*-value < 0.05 was considered statistically significant. Statistical analyses used are specified in Figure legends.

## 3. Results

### 3.1. Generation of a CD8 T Cell-Reactive Mouse Melanoma Model Sensitive to PD1 Inhibition

Based on the importance of tumor MHC-I expression and intact IFNγ signaling for clinical response to single agent anti-PD1 immunotherapy [13,14,26], we designed a rational workflow to generate a transplantable mouse melanoma model responsive to PD1 blockade. The workflow included (i) selection of a mouse melanoma parental cell line responsive to exogenous IFNγ, including induction of MHC-I and PD-L1 expression, (ii) augmentation of tumor mutation burden through ultraviolet (UVR)-mutagenesis, (iii) post-UVR single-cell cloning, (iv) selection of immunogenic tumor clones through indirect CD8 T-cell recognition test, (v) in vivo tumor engraftment, and vi) evaluation of local and systemic immunological responses, including systemic memory.

**Figure 1:**
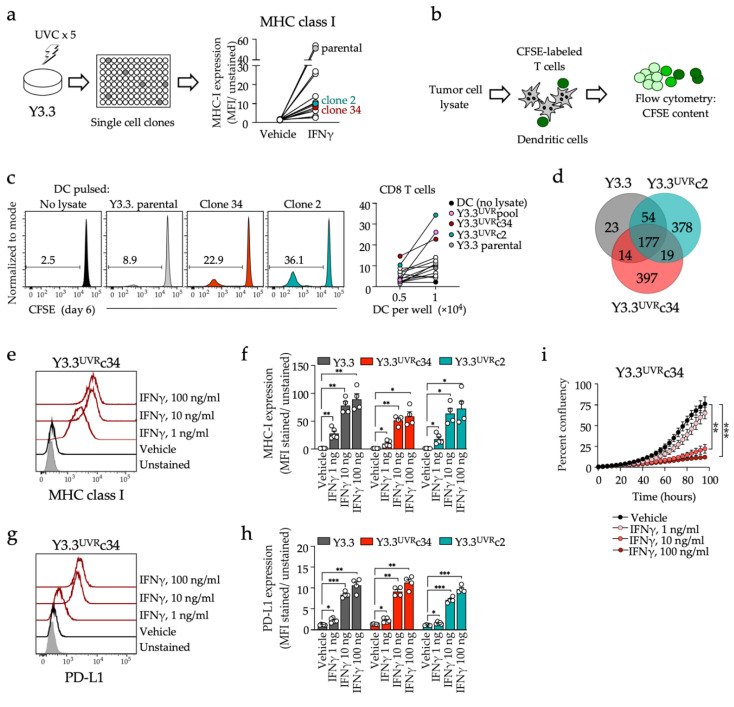
Generation of cell lines and analysis of immunotherapy response biomarkers. (**a**) Y3.3 cells were subjected to five rounds of UV irradiation followed by single cell cloning. Clones were tested for baseline and IFNγ-induced MHC-I expression. The right panel shows a summary of MHC-I expression for eleven selected clones and parental Y3.3 cells. (**b**) Immunoreactivity test. Bone marrow-derived dendritic cells were pulsed with tumor cell lysates and co-cultured with CFSE-labeled syngeneic T cells. T-cell proliferation was assessed by flow cytometry as a decrease in CFSE fluorescence. (**c**) Representative examples of CFSE dilution with T cell:DC ratio 10:1 (left panels) and means of triplicates (right panel). Y3.3^UVR^pool, combined tumor lysates from multiple clones. (**d**) Number and distribution of missense mutations in Y3.3, Y3.3^UVR^c2, and Y3.3^UVR^c34. (**e**–**h**) Expression of MHC-I (**e**,**f**) and PD-L1 (**g**,**h**) after 24 h IFNγ exposure, analyzed by flow cytometry. Representative examples (**e**,**g**) and summaries (**f**,**h**) are shown. (**i**) Proliferation of Y3.3^UVR^c34 cells exposed to vehicle or IFNγ (IncuCyte confluency, mean ± SD, 4 images per treatment per time point). Data in (**f**,**h**,**i**) were compared using one-way ANOVA with Dunnett’s multiple comparisons test (* *p* < 0.05, ** *p* < 0.01; *** *p* < 0.001).

From the panel of four YUMM (Y) cell lines [15], Y3.3 cells were selected for further manipulation based on their superior response to IFNγ with upregulation of the two key immunotherapy response biomarkers, MHC-I and PD-L1 (Figure S1a,b). This pattern was similar to YUMM-derived immunogenic YUMMER(YR).G model [22] but different from the commonly used YR1.7 cells [21] that showed reduced MHC-I expression compared to parental cells (Figure S1c) and no PD-L1 expression upon IFNγ induction (Figure S1d). MHC-I expression of YR1.7 cells was heterogeneous due to the presence of both positive and negative subclones (Figure S1e). To increase immunogenicity, Y3.3 cells were subjected to UV irradiation followed by single cell cloning (Figure 1a). The resulting Y3.3^UVR^ clones were selected for immune reactivity by assessing the proliferation of syngeneic CD8 T cells labeled with a cell division tracker and co-cultured with syngeneic bone-marrow-derived dendritic cells pulsed with tumor cell lysates (Figure 1b,c), and also tested for IFNγ-mediated induction of MHC-I expression (Figure 1a). Two clones (c2 and c34) demonstrated high immune reactivity, comparable to the immunogenic models YR1.7 and YR.G (Figure S1f) while retaining IFNγ-mediated induction of MHC-I expression. Whole-exome sequencing confirmed that both clones had accumulated additional mutations compared with parental cells, with 628 and 607 somatic missense mutations identified for Y3.3^UVR^c2 and Y3.3^UVR^c34, respectively (Figure 1d and Table 1). Parental Y3.3, Y3.3^UVR^c2 and Y3.3^UVR^c34 cells exhibited similar in vitro morphology and growth rates (Figure S1g,h) and responded to increasing doses of IFNγ by upregulating MHC-I and PD-L1 in a dose-dependent manner (Figure 1e–h and Figure S1i). Growth of Y3.3^UVR^c34 cells was inhibited by IFNγ in a dose-dependent manner (Figure 1i), without an overall increase in cell death.

### 3.2. Immunotherapy Response and Protective Memory

**Figure 2:**
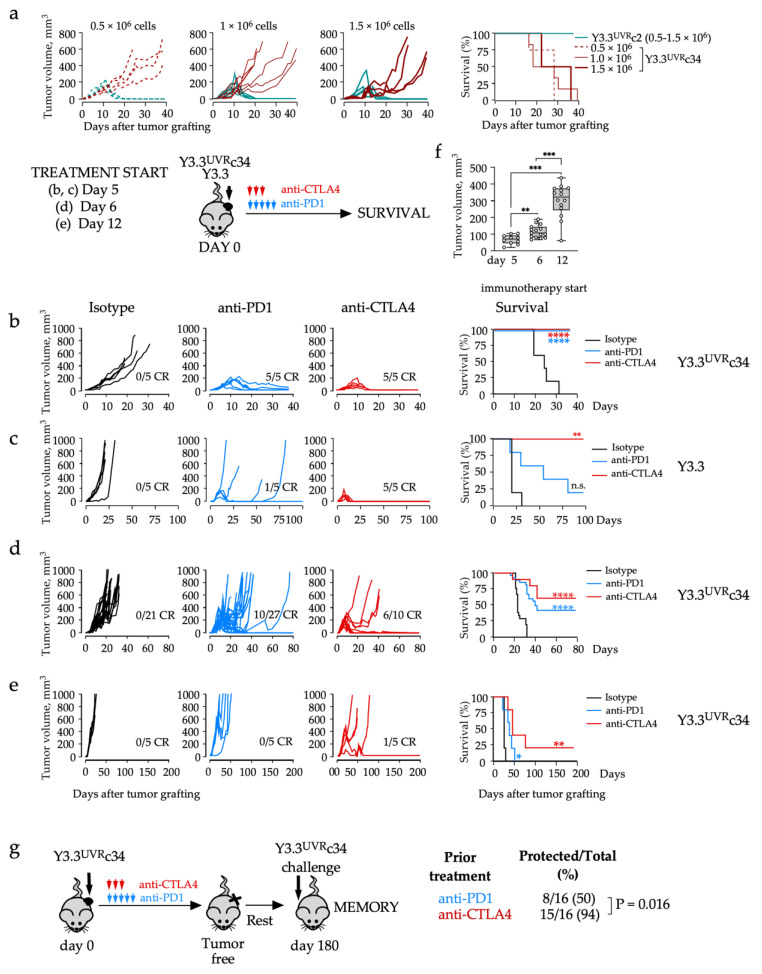
In vivo tumor growth and immunotherapy response of Y3.3^UVR^ clones. (**a**) Y3.3^UVR^c2 or Y3.3^UVR^c34 cells at indicated dose per animal were injected subcutaneously into the flank of female C57BL/6 hosts. Tumor growth (**left** panels) and animal survival (**right** panel) were monitored. (**b**–**e**) Y3.3^UVR^c34 or parental Y3.3 cells, as indicated, were injected subcutaneously into female C57BL/6 hosts. Immunotherapy with anti-PD1 (0.3 mg/mouse × 5 to the total of 1.5 mg/mouse, blue), or anti-CTLA4 (0.2 mg × 1 then 0.1 mg/mouse × 2 to the total of 0.4 mg/mouse, red) was initiated on day 5 (**b**,**c**), day 6 (**d**) or day 12 (**e**). Left panels, tumor growth; right panels, animal survival (* *p* < 0.05, ** *p* < 0.01, **** *p* < 0.0001, n.s., not significant, Mantel-Cox test). Data in (**d**) represent a summary of three independent experiments. Complete responses (CR) and group sizes are indicated. (**f**) Tumor volume at the start of treatment (** *p* < 0.01, *** *p* < 0.001, Mann–Whitney test). (**g**) Test for tumor-specific memory. Animals that had rejected Y3.3^UVR^c34 tumors after treatment with anti-PD1 or anti-CTLA4, were rested and re-challenged with Y3.3^UVR^c34 cells into a contralateral flank, without additional treatment. Numbers indicate tumor-free/total animals at completion. Data were derived from four separate experiments and compared using Fisher’s exact test.

When subcutaneously grafted into sex-matched (female) immunocompetent syngeneic recipients, Y3.3^UVR^c34 tumors progressed over the 40-day study period, while all Y3.3^UVR^c2 tumors were spontaneously rejected after reaching an average size of 173 ± 67mm^3^ (Figure 2a). These patterns were reminiscent of YR tumors that either progressed (YR1.7) or were spontaneously rejected (YR.G) in sex-matched recipients, as expected (Figure S2a–c). We next tested immunotherapy response in mice grafted with subcutaneous Y3.3^UVR^c34 tumors (Figure 2b,d,e). Anti-PD1 or anti-CTLA4 therapy was initiated 5 days after tumor grafting, fully controlled tumor growth (Figure 2b), while parental Y3.3 tumors were controlled by anti-CTLA4, but not anti-PD1 treatment (Figure 2c). A delay in immunotherapy reduced treatment efficacy (Figure 2d,e); however, this could be overcome by combination (anti-CTLA4 plus anti-PD1) treatment (Figure S2e). A decrease in treatment efficacy was consistent with a higher tumor burden at the start of immunotherapy (Figure 2f).

Incremental increase in anti-CTLA4 (from 0.4 mg to 0.6 mg cumulative dose) increased complete response (CR) rates from 60% (Figure 2d) to 100% (Figure S2h). However, anti-PD1 efficacy did not change with an increase in drug dose (Figure S2i). Finally, immunotherapy resulted in the development of protective memory in surviving animals. Upon re-challenge with Y3.3^UVR^c34 cells, protection was 50% and 94% for anti-PD1 and anti-CTLA4 groups, respectively (Figure 2g).

In summary, Y3.3^UVR^c2 tumors were spontaneously rejected while Y3.3^UVR^c34 tumors were controlled with immunotherapy. Combined immunotherapy or anti-CTLA4 dose escalation afforded improved tumor control. Furthermore, immunotherapy resulted in the development of systemic immunological memory that protected animals from tumor re-challenge.

### 3.3. Local T-Cell Recruitment and PD1 Checkpoint Engagement

**Figure 3:**
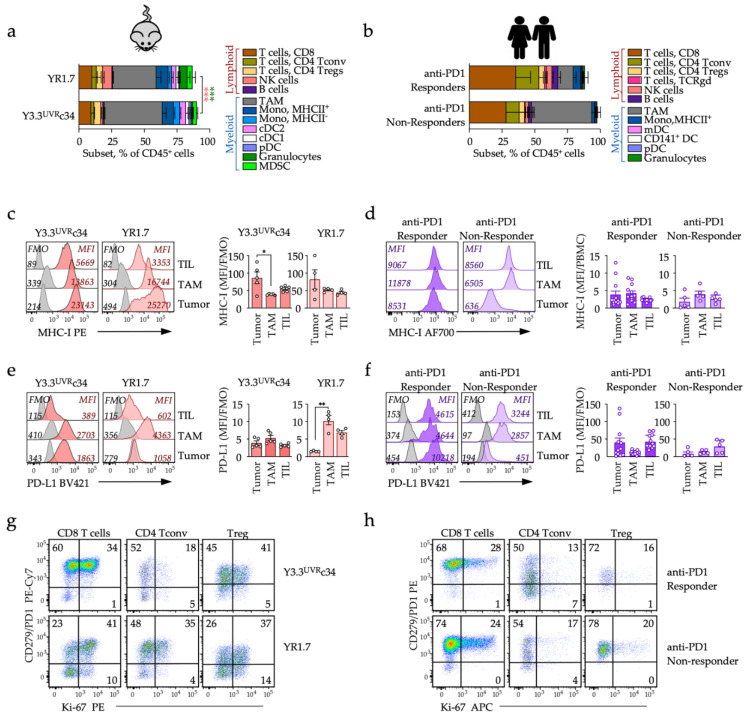
Comparative immune contexture and phenotype of mouse and human melanoma. Immune contexture (**a**,**b**) and phenotype of tumor cells (**c**–**f**) and tumor-infiltrating T cells (**g**–**h**) in mouse (**a**,**c**,**e**,**g**) or human tumors (**b**,**d**,**f**,**h**). Endpoint YR1.7 and Y3.3^UVR^c34 mouse tumors (from sex-matched, untreated animals), or human biopsies obtained from patients with known anti-PD-1 responder status prior to immunotherapy start, were enzymatically processed, cryopreserved and bulk-analyzed by flow cytometry. (**a**) Immune contexture of YR1.7 (*n* = 4) and Y3.3^UVR^c34 (*n* = 6) tumors (*** *p* < 0.001, unpaired *t*-test), analyzed as shown in Figure S3a,b. (**b**) Immune contexture of human tumors, analyzed as reported in [13]. (**c**,**d**) MHC-I expression on melanoma cells (Tumor), macrophages (TAM) and T cells (TIL) in mouse (**c**) and human melanoma (**d**). Representative examples (left panels) and summaries (right panels). Expression was calculated as a ratio of fluorescence intensity in the relevant channel (MFI, color-filled histograms) over the background fluorescence (FMO, gray-filled histograms) for mouse tumors, or as MFI of the population shown over MFI of control blood mononuclear cells (PBMC) for human tumors, as reported in [13]. MFI and FMO values are shown. * *p* < 0.05, one-way ANOVA with Tukey’s multiple comparisons test. (**e**,**f**) PD-L1 expression on melanoma cells (Tumor), macrophages (TAM) and T cells (TIL) in mouse (**e**) and human melanoma (**f**). Representative examples (**left** panels) and summaries (**right** panels). Expression was calculated as a ratio of fluorescence intensity in the relevant channel (MFI, color-filled histograms) over the background fluorescence (FMO, gray-filled histograms). MFI and FMO values are shown. ** *p* < 0.01, one-way ANOVA with Tukey’s multiple comparisons test. (**g**,**h**) Phenotype of tumor-infiltrating CD8 T cells, conventional CD4 T cells (Tconv) and regulatory T cells (Treg) in mouse (**g**) and human tumors (**h**). Expression of PD-1 and subset proliferation (Ki-67) are shown. Numbers indicate frequency of cells in relevant gates.

In order to gain insights into the local changes associated with tumor control, we employed multiparameter flow cytometric analysis of tumor microenvironment (TME) (Figure 3a and Figure S3). Side-by-side comparison of large endpoint Y3.3^UVR^c34 and YR1.7 tumors identified thirteen immune infiltrating subsets with a myeloid prevalence in both models, including tumor-associated macrophages (TAM), MHC-II^−^ and MHC-II^+^ monocyte subsets, granulocytes (significantly enriched in YR1.7 model), myeloid-derived suppressor cells (MDSC) and three populations of dendritic cells (DCs) including plasmacytoid DCs (pDCs), conventional CD103^+^ DCs (cDC1) and conventional CD11b^+^ DCs (cDC2) (Figure 3a and Figure S3a). Among lymphoid cells, there were few B cells while natural killer (NK) cells were significantly enriched in YR1.7 tumors; among tumor-infiltrating T-lymphocytes (TILs), CD8 T cells, conventional CD4^+^Foxp3^−^ T cells (Tconv) and CD4^+^Foxp3^+^CD25^+^ regulatory T cells (Tregs) were present at similar frequencies in both models (Figure 3a and Figure S3b). Tumor myeloid predominance was established early and maintained over tumor growth period (Figure S3c). Analysis of Y3.3^UVR^c34 tumors collected early (day 5, average tumor volume 39 mm^3^), mid-experiment (day 10, tumor volume 206 mm^3^), and at endpoint (day 20–21, tumor volume 1083 mm^3^), revealed that all major myeloid and lymphoid subsets were present throughout, but their relative proportion changed over time reflecting tumor evolution. In the myeloid compartment, there was a gradual decrease in MDSC and a progressive increase in macrophages and MHC-II^+^ monocytes (Figure S3c). In the lymphoid compartment, there was a steady increase in CD8 and Treg cells, and a temporary early increase in NK cells (Figure S3c).

We next compared tumor contexture of murine tumors to human melanoma biopsies collected prior to the start of anti-PD1 monotherapy [13]. Human melanoma biopsies, processed and analyzed similarly to mouse tumors, revealed higher lymphoid content and lymphoid/myeloid ratio (8.1 ± 2.1) than mouse melanoma (0.28 ± 0.02 for Y3.3^UVR^c34 and 0.43 ± 0.1 for YR1.7; Figure 3a,b). When corrected for response status and after exclusion of lymphoid tissue metastases, lymphoid/myeloid ratio in human non-lymph node metastases was higher in anti-PD1 responders (7.6 ± 9.4) than anti-PD1 non-responders (1.4 ± 1.2), indicating that innate immunotherapy resistance is associated with myeloid prevalence (Figure 3b). Phenotype of mouse melanoma cells was consistent with a local IFNγ response, with high tumor MHC-I expression comparable to TME in both Y3.3^UVR^c34 and YR1.7 models (Figure 3c), similar to the pattern observed in anti-PD1 responding patients (Figure 3d), while tumor MHC-I was frequently downregulated in non-responding patients (Figure 3d and [13]). Unlike MHC-I, PD-L1 was expressed on Y3.3^UVR^c34 but notably absent on YR1.7 tumor cells (Figure 3e), consistent with the lack of PD-L1 expression on YR1.7 cells exposed to IFNγ in vitro (Figure 1a); PD-L1 was expressed in the TME of both models. PD-L1 expression in human tumors mirrored MHC-I expression pattern (Figure 3f), consistent with a strong correlation between the two markers [13]. Finally, all mouse and human tumors contained exhausted PD1^+/high^ T cells. The fraction of exhausted CD8 T cells was enriched in Y3.3^UVR^c34 compared to YR1.7 tumors (Figure 3g) and consistent with high PD-1 expression on human TILs (Figure 3h).

Thus, microenvironment of YR1.7 and Y3.3^UVR^c34 tumors showed myeloid predominance, in contrast to human tumors. Both the Y3.3^UVR^c34 mouse and anti-PD1 responding human tumors showed infiltration with tumor-specific CD8 T cells exhibiting high expression of the PD1 inhibitory checkpoint, while its ligand PD-L1 was highly expressed on both tumor cells and TME.

### 3.4. Distinct Early Changes in Immune Phenotypes during Checkpoint Blockade

**Figure 4:**
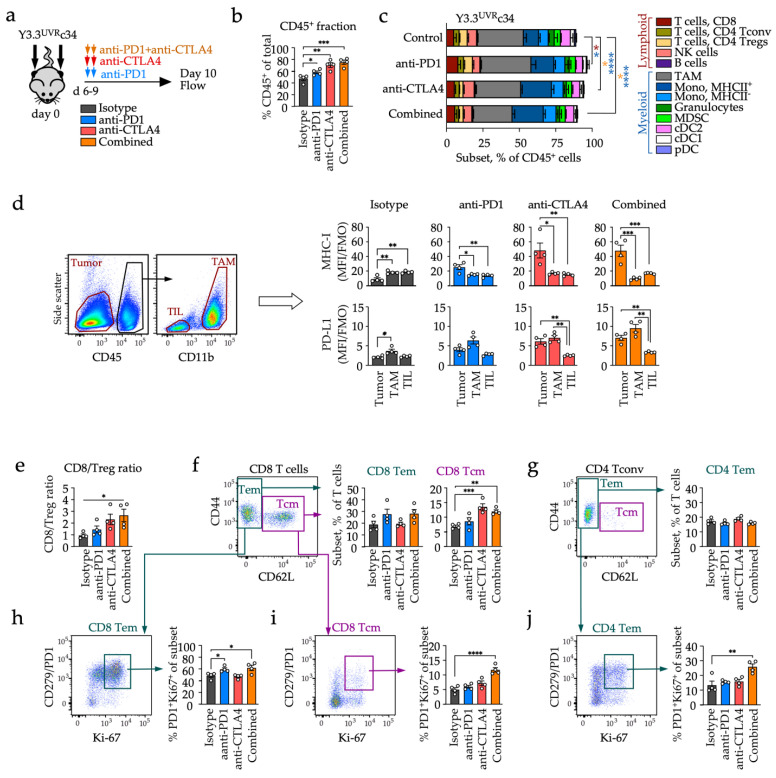
Changes in immune contexture and tumor phenotype early on-therapy. (**a**) Experimental set-up. Flow cytometric analyses were performed on samples collected after two rounds of immunotherapy. (**b**) CD45 (immune) infiltration in isotype control (gray), anti-PD1 (blue), anti-CTLA4 (red) or combined (anti-PD1 plus anti-CTLA4, orange) treated groups. (**c**) Tumor immune contexture was analyzed as shown in Figure S3a,b. Significant comparisons are indicated. (**d**) MHC-I and PD-L1 expression on tumor cells (Tumor), macrophages (TAM) and T cells (TIL). Left panels, representative flow gating for tumor cells, TIL and TAM subsets; right panels, MHC-I (**top** row) and PD-L1 expression (**bottom** row) in different treatment groups. (**e**) CD8/Treg ratio; (**f**,**g**) T-cell phenotypes. Representative examples (**left** panels) and a summary (**right** panels) of effector/memory (Tem) and central memory (Tcm) phenotypes for CD8 (**f**) and CD4 T conventional populations (**g**). (**h**–**j**) Co-expression of the inhibitory receptor PD1 and proliferation marker Ki-67 for each of the T-cell phenotypes, as indicated. For all group comparisons, one-way ANOVA with Tukey’s multiple comparisons test was used (* *p* < 0.05; ** *p* < 0.01; *** *p* < 0.001; **** *p* < 0.0001).

We next characterized TME in animals receiving immunotherapy. Early on-treatment changes observed after two cycles of either anti-PD1, anti-CTLA4 or combined immunotherapy, in animals grafted with bilateral tumors (Figure 4a), demonstrated a significant increase in immune infiltration (Figure 4b), with significant enrichment in MHC-II^+^ monocytes across all immunotherapy-treated groups (Figure 4c). In addition, anti-PD1 treatment was associated with an increase in CD8 T cells, while anti-CTLA4 and combined treatment led to a decrease in regulatory T cells (Figure 4c). There was an increase in MHC-I and PD-L1 expression on tumor cells, particularly in the anti-CTLA4 and combined immunotherapy cohorts (Figure 4d and Figure S4). MHC-I expression on tumor cells was significantly above the TME levels in all immunotherapy-treated groups, opposite to control (Figure 4d, top row). PD-L1 expression was highly elevated on both tumor cells and macrophages in animals receiving anti-CTLA4 or combined immunotherapy (Figure 4d, bottom row).

To directly compare T-cell activation across treatment groups, we performed a detailed phenotypic analysis of TILs (Figure 4e–j). The CD8/Treg ratio showed an upward trend for anti-CTLA4-based therapies (Figure 4e), consistent with the anti-CTLA4 mediated decrease in Treg frequencies (Figure 4c). CD8 T cells exhibited either a CD44^high^CD62L^−^ effector/memory (Tem) or a CD44^high^CD62L^+^ central memory (Tcm) phenotype (Figure 4f), whereas CD4 Tconv were almost exclusively CD44^high^CD62L^−^ effector cells (Figure 4g). Immunotherapy did not significantly change CD8 Tem content, although there was an upward trend for anti-PD1-treated groups, while Tcm-like CD8 cells were significantly elevated in the two groups that received anti-CTLA4 (Figure 4f, right panels); there were no changes in the CD4 Tem content (Figure 4g). As we had no other means of tracking antigen-specific T cells, PD1 expression was used as an indicator of tumor reactivity [27]. A fraction of PD1^+^ CD8 Tem cells was significantly elevated in animals that received anti-PD1 or combined treatment (not shown), as was the proliferation of this cell subset (Figure 4h). There was also an increase in proliferating PD1^+^ CD8 Tcm and PD1^+^ CD4 Tem on combination treatment (Figure 4i–j).

Taken together, immune activation in immunotherapy-responding tumors is reflected in characteristic changes in tumor immune contexture, increased expression of antigen-presenting molecules and immune checkpoints on both tumor and myeloid cells, and increased T-cell reactivity in both CD8 and CD4 compartments, with the highest magnitude of changes observed following combined (anti-PD1 plus anti-CTLA4) immunotherapy.

### 3.5. Distinct Effects of Anti-PD1 and Anti-CTLA4 on Circulating T Cell Subsets

**Figure 5:**
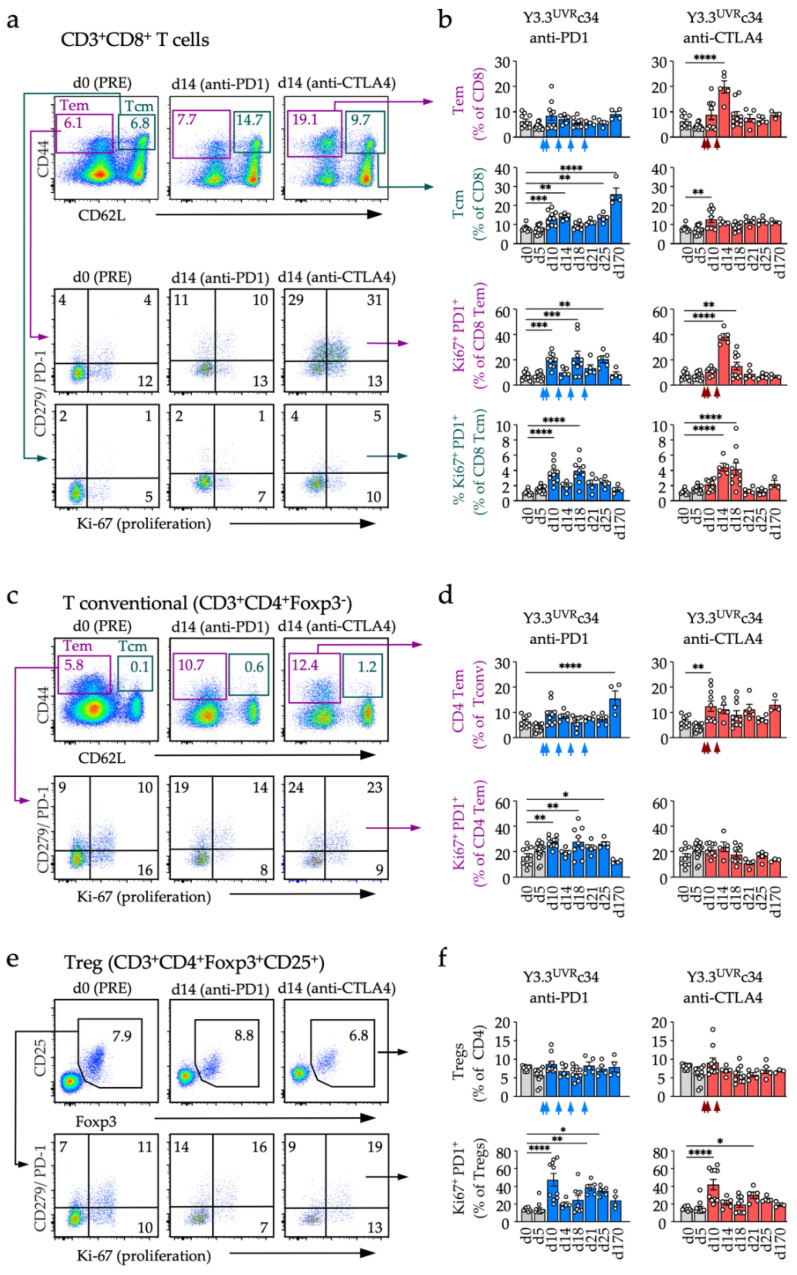
Changes in circulating T-cell subsets during immunotherapy response. Animals grafted with Y3.3^UVR^c34 tumors were bled on the day of (but prior to) tumor cell injection (PRE, day 0) and at indicated times after tumor inoculation; frequencies and phenotypes of circulating T cells were assessed by flow cytometry. (**a**,**b**) Frequency of CD8 Tem (CD44^+^CD62L^−^, purple) and Tcm (CD44^+^CD62L^+^, teal) among CD8 T cells (**a**, top row; **b**, first and second row). PD1 and Ki-67 co-expression in CD8 Tem cells (**a**, second row; **b**, third row) and Tcm cells (**a**, third row; **b**, fourth row). Representative scatter plots from anti-PD1or anti-CTLA4 treated animals (**a**) and summary data (**b**) are shown. (**c**,**d**) As above for (**a**,**b**) but conventional (CD4^+^Foxp3^−^) T cells were analyzed for Tem frequency (**top** rows) and PD1/Ki-67 co-expression (**bottom** rows). (**e**,**f**) Frequency of Tregs (CD4^+^Foxp3^+^) among CD4 T cells (**top** row), and PD1/Ki-67 co-expression (**bottom** row). Data were collated from 2–4 independent experiments. Arrows indicate drug delivery. The timeline indicates days after tumor cell injection on day 0. Significant comparisons with PRE values are shown (one-way ANOVA with Tukey’s multiple comparisons test; * *p* < 0.05; ** *p* < 0.01; *** *p* < 0.001; **** *p* < 0.0001).

Recent data highlighted the importance of activating novel T-cell clonotypes, including the recruitment of new specificities from blood, for not only anti-CTLA4 but also anti-PD1 response [28]. We therefore tracked changes in circulating effector and memory T-cell subsets and accompanying phenotypic changes associated with anti-PD1 or anti-CTLA4 mediated tumor control (Figure 5). These experiments included multiple animals from independent experiments, although we note that over time the numbers of animals decrease due to the termination of progressing animals. Anti-CTLA4 treatment was associated with a coordinated “spike” in effector/memory (CD44^high^CD62L^−^) CD8 T cells around day 14 (Figure 5a,b top row), following treatment completion (day 12) and coinciding with the start of tumor rejection. In contrast, anti-PD1 treatment led to an increase in central memory type (CD44^high^CD62L^+^) CD8 Tcm cells from day 10 onwards (Figure 5a top row and Figure 5b second row). This accumulation of memory T cells was associated with an increase in proliferating, PD1 expressing cells with a corresponding (Tem or Tcm) phenotype that occurred on days 10–18, including a profound increase in PD1^+^Ki-67^+^ CD8 Tem on day 14, corresponding to the Tem “spike” described earlier (Figure 5a second and third row, and Figure 5b third and fourth row). In the CD4 (Tconv) compartment, Tcm frequencies remained very low but effector/memory phenotype (CD44^high^CD62L^−^) Tconv accumulated during the late memory phase (day 170), while their proliferation slowed (Figure 5c,d). Regulatory (Treg) cells in all experimental groups responded with rebound proliferation to tumor grafting (day 10–25), while total Treg frequencies did not change over time (Figure 5e,f). PD1 expression remained elevated on all T-cell subsets in both treatment groups, while proliferation was variable (Figure S5a–d).

Thus, we observed therapy-specific differences in circulating T-cell subsets, with anti-CTLA4 significantly elevating blood CD8 and CD4 effector T cells early in treatment and anti-PD1, resulting in a slower response. An early expansion of effector CD8 T cells is similar to that observed in human anti-PD1 responder cohorts following immunotherapy therapy start [9]. 

## 4. Discussion

We report a rational workflow to generate a transplantable mouse melanoma model responsive to anti-PD1 monotherapy, based on information obtained from both human and animal studies. While high tumor mutation burden is associated with better immunotherapy response through the generation of neoantigens that establish tumor “foreignness” to the immune system [3,21], CD8 T-cell recognition of these neoantigens requires tumor MHC-I expression [10] and intact IFNγ signaling [11]. IFNγ increases neoantigen presentation via MHC-I upregulation but also drives adaptive immune resistance by enhancing tumor PD-L1 expression and T-cell exhaustion [29]. The efficacy of PD1 blockade is reliant upon disrupting interactions between PD1-positive T cells and PD-L1-positive melanoma cells [29,30], ultimately leading to the expansion of tumor neoantigen-specific clones and tumor control. Among the various YUMM-derived models tested for MHC-I and PD-L1 expression, only Y3.3 cells upregulated both MHC-I and PD-L1 upon IFNγ exposure and were thus selected for this study. Y2.1 cells were phenotypically MHC-I deficient, raising mechanistic questions with regard to their responsiveness to PD1 blockade in vivo [17]. YR1.7 cells failed to upregulate PD-L1 in response to IFNγ while YR1.7 tumors retained PD-L1-null phenotype, suggesting the dominant role of stroma- and TME-restricted PD-L1 for anti-PD1 response in that model [21,31] and consistent with an impeded growth of YR1.7 tumors in mice lacking PD-L1 expression in non-hematopoietic cells [32]. Additionally, the inherent heterogeneity of YR1.7 line—likely due to genetic drift—may underlie discrepancies in tumor phenotypes and immunotherapy responses in different reports [20,21,31]. We next employed UVR-mutagenesis to increase tumor mutation burden [21], followed by single-cell cloning to reduce tumor heterogeneity, in order to minimize antigenic drift that underlies tumor immune escape [33]. The two selected clones (c2 and c34) demonstrated indirect immune reactivity comparable to immunotherapy-sensitive YR1.7 and spontaneously controlled YR.G models, consistent with a comparable tumor mutation burden [21,22]. Y3.3^UVR^c34 tumors were responsive to anti-PD1 and/or anti-CTLA4, with a better response rate achieved with earlier drug administration or higher dose of anti-CTLA4, while only combined (anti-PD1 plus anti-CTLA4) therapy was effective against larger established tumors. This is in line with human studies linking higher tumor burden with treatment failure [34] providing a rationale for first-line combination treatment in high volume disease.

Side-by-side comparison of tumors responding to anti-PD1 versus anti-CTLA4, showed differences in response kinetics and distinct changes in response biomarkers, both local (tumor contexture) and systemic (circulating T-cell subsets). Response to anti-CTLA4 was rapid, with visible tumor growth arrest observed between the second and third treatment cycles in most animals, compared to the fourth and fifth cycles for anti-PD1. Early on-therapy changes such as upregulation of MHC-I and PD-L1 on melanoma cells and in the microenvironment, were consistent with T-cell activation and local IFNγ release [7]. Early tumor influx of MHC-II-positive monocytes observed in all treatment groups, is in line with previously reported association between an increase in blood MHC-II-positive monocytes and improved responses to anti-PD1 in patients with metastatic melanoma [35]; after recruitment into tumors, such monocytes can act as antigen-presenting cells directly, differentiate into inflammatory CD11c-high tumor-associated macrophages that favor T-cell recruitment and activation [36], and/or differentiate into dendritic cells, enhancing antigen presentation and consequently local immune recognition [37]. Combined immunotherapy was associated with superior T-cell activation and improved CD8/Treg ratio, consistent with efficacy against large established tumors. Further studies are required to decipher the exact mechanism of synergy between PD1 and CTLA4 blockade. Although elimination of intratumoral Tregs by anti-CTLA4 may play a role [38,39], we saw neither complete depletion of intratumoral Tregs nor change in systemic Treg numbers. This is broadly consistent with a selective depletion of tumor-specific Tregs observed in transgenic adoptive transfer models, with a marginal effect on endogenous non-transgenic Treg populations [38].

Finally, anti-PD1 and anti-CTLA4 treatments had disparate effects on circulating immune cells. CTLA4 blockade produced a distinctive spike in effector T cells that coincided with the onset of rejection, while PD1 blockade lacked this distinctive increase, showing instead a smaller magnitude, gradual increase in central memory-like CD8 T cells. Changes in circulating subsets need to be confirmed, as sequential samplings are affected by attrition of non-responding animals and ethical limitations to sampling frequency. A more detailed analysis of T-cell clonality (via sorting and TCR sequencing) will confirm whether tumor control is predominantly mono- or oligoclonal, which may have implications for the emergence of tumor escape variants and late progression. In human melanoma, superior effector CD8 T-cell expansion is achieved with combination immunotherapy as compared to anti-PD1 monotherapy and correlates with improved outcomes [40]. While this study focused on the effect of anti-PD1 monotherapy and we chose anti-CTLA-4 monotherapy as a comparator, we also observed a superior effect of combined immunotherapy in high-volume disease. Further investigation is warranted into the local and systemic effects of combined immunotherapy, in particular, the establishment of immunological memory as a correlate of durable protection. Since both monotherapies generated immunological memory in our study, we propose that combined immunotherapy helps generate phenotypically and likely, functionally non-redundant circulating memory cells, in addition to resident memory described elsewhere [41]. Our use of a different site for the memory challenge highlights the role of systemic recirculating memory in durable protection against metastatic disease, as opposed to resident memory T cell populations, whose role was studied extensively in animal models [41,42]. Furthermore, the occasional emergence of late progressing tumors in our model is consistent with long-term immunological control or immune surveillance. While the emergence of late-progressing tumors is clearly due to the failure of immune surveillance mechanisms, it is unclear whether this could be related to tumor adaptation to the high interferon environment, such as the acquisition of sustained intrinsic interferon signaling [29].

In summary, we have established two related mouse melanoma models, one yielding progressing tumors that responded to both single-agent or combined immunotherapy, the other resulting in spontaneously regressing tumors. The two models display important immunotherapy response biomarkers, will be valuable for studying the molecular mechanisms underlying immunotherapy resistance, and may aid in the selection of salvage therapies for immunotherapy-resistant patients.

## 5. Conclusions

In this study, we have established a biomarker-driven workflow to generate a transplantable mouse melanoma model responsive to single-agent immune checkpoint blockade. This model displayed immunotherapy response biomarkers, including tumor expression of MHC-I and PD-L1, immune cell expression of the PD-1 checkpoint, tumor cells’ responsiveness to IFNγ, high tumor mutation burden, low tumor heterogeneity, and high immune reactivity in vitro and in vivo. Tumors established in mice phenotypically resembled human tumors, and tumor-bearing animals differentially responded to single agent- and combined immunotherapy. These responses were correlated with phenotypic and qualitative changes in tumor cells, immune infiltrates, and circulating T-lymphocyte subsets and were linked to protective memory. Our model provides unique insights into the discrete immunological mechanisms underlying the durability of response to the two most clinically relevant checkpoint blockers and could aid in the selection of salvage therapies for immunotherapy-resistant tumors.

## Figures and Tables

**Table 1:** Mutations in Y3.3^UVR^ models based on whole exome sequencing.

Mutations	Y3.3	Y3.3^UVR^c2	Y3.3^UVR^c34
Somatic missense mutations	268	628	607
*Braf*	V600E	V600E	V600E
*Pten*	Intact	Intact	Intact
*Cdnk2a*	Deleted	Deleted	Deleted

## Data Availability

Data supporting the findings of this study are provided as supplementary data or available from the corresponding author upon reasonable request.

## Supplementary Materials

The following supporting information can be downloaded at: https://www.mdpi.com/article/10.3390/cancers14194830/s1, Figure S1: Immune phenotype and immune reactivity of selected YUMM and YUMMER cells lines; Figure S2: Growth and immunotherapy response patterns of selected YUMM-derived tumors; Figure S3: Flow cytometric evaluation of tumor immune contexture; Figure S4: Early-treatment changes in tumor cell phenotype; Figure S5: Changes in circulating T-cell phenotypes following immunotherapy with anti-PD1 or anti-CTLA4 antibodies; Table S1: Antibodies and reagents for flow cytometry.
