# Supplementary material for: Durable Responses to Anti-PD1 and Anti-CTLA4 in a Preclinical Model of Melanoma Displaying Key Immunotherapy Response Biomarkers
Source: Cancers (Basel). 2022 Oct 3;14(19):4830. doi: 10.3390/cancers14194830 (PMC9564179; doi:10.3390/cancers14194830)
Supplement: Supplementary file 1 [file cancers-14-04830-s001.zip › cancers-1895743 Supplementary.pdf]

cancers

MDPI

Durable Responses to Anti-PD1 and Anti-CTLA4 in a Preclinical Model of Melanoma Displaying Key Immunotherapy Response Biomarkers

Supplementary data

Supplementary Figures

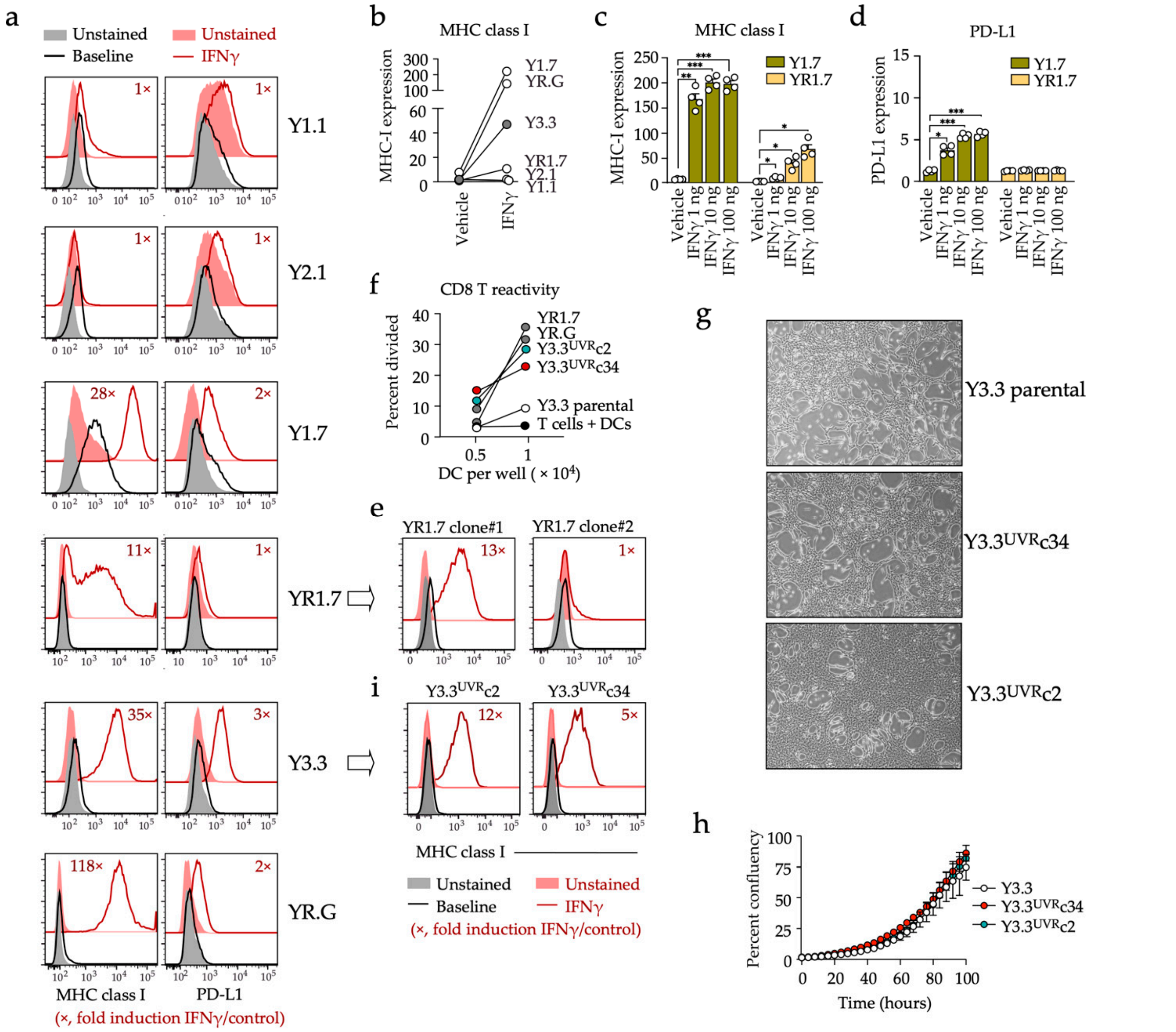

Figure S1. Immune phenotype and immune reactivity of selected YUMM and YUMMER cells lines. (**a**) Expression of MHC-I (left panels) and PD-L1 (right panels) on indicated YUMM (Y) and YUMMER (YR) cell lines as assessed by flow cytometry at baseline (solid black lines) and 24 hours after exposure to 1ng/ml IFNγ (solid red lines). Gray-shaded and red-shaded histograms indicate the respective background fluorescence (LiveDead-only stained samples). Numbers indicate fold induction in marker expression (IFNγ/baseline). (**b**) MHC-I expression on various Y and YR cell lines, 24 hours after exposure to 1ng/ml IFNγ (means of biological triplicates). (**c**,**d**) Expression of MHC-I (c) and PD-L1 (d) on YR1.7 and parental Y1.7 cells at baseline and after 24-hour IFNγ exposure (**p* < 0.05; ** $p < 0.01$; *** $p < 0.001$, one-way ANOVA with Dunnett's post-test). (**e**) Differential expression of MHC-I on two YR1.7-derived single cell clones, at baseline and 24 hours after exposure to 1ng/ml IFNγ. (**f**) Comparison of CD8 T-cell reactivity for Y3.3^{UVR}c34, Y3.3^{UVR}c2, YR1.7 and YR.G, in the test

described in Figure 1b and 1c. (**g**) Morphology of parental Y3.3 cells and UVR derivatives (magnification 40×, scale bar 500mm). (**h**) Representative proliferation curves of Y3.3, Y3.3^{UVR}c2 and Y3.3^{UVR}c34 cells measured as percent confluence (IncuCyte imaging) every 4h for up to 96h. Data shown as mean ± SD (4 images per treatment per time point, representative of two independent experiments). (**i**) Examples of MHC-I expression on Y3.3^{UVR}c2 and Y3.3^{UVR}c34 cells, at baseline and 24 hours after exposure to 1ng/ml IFNγ.

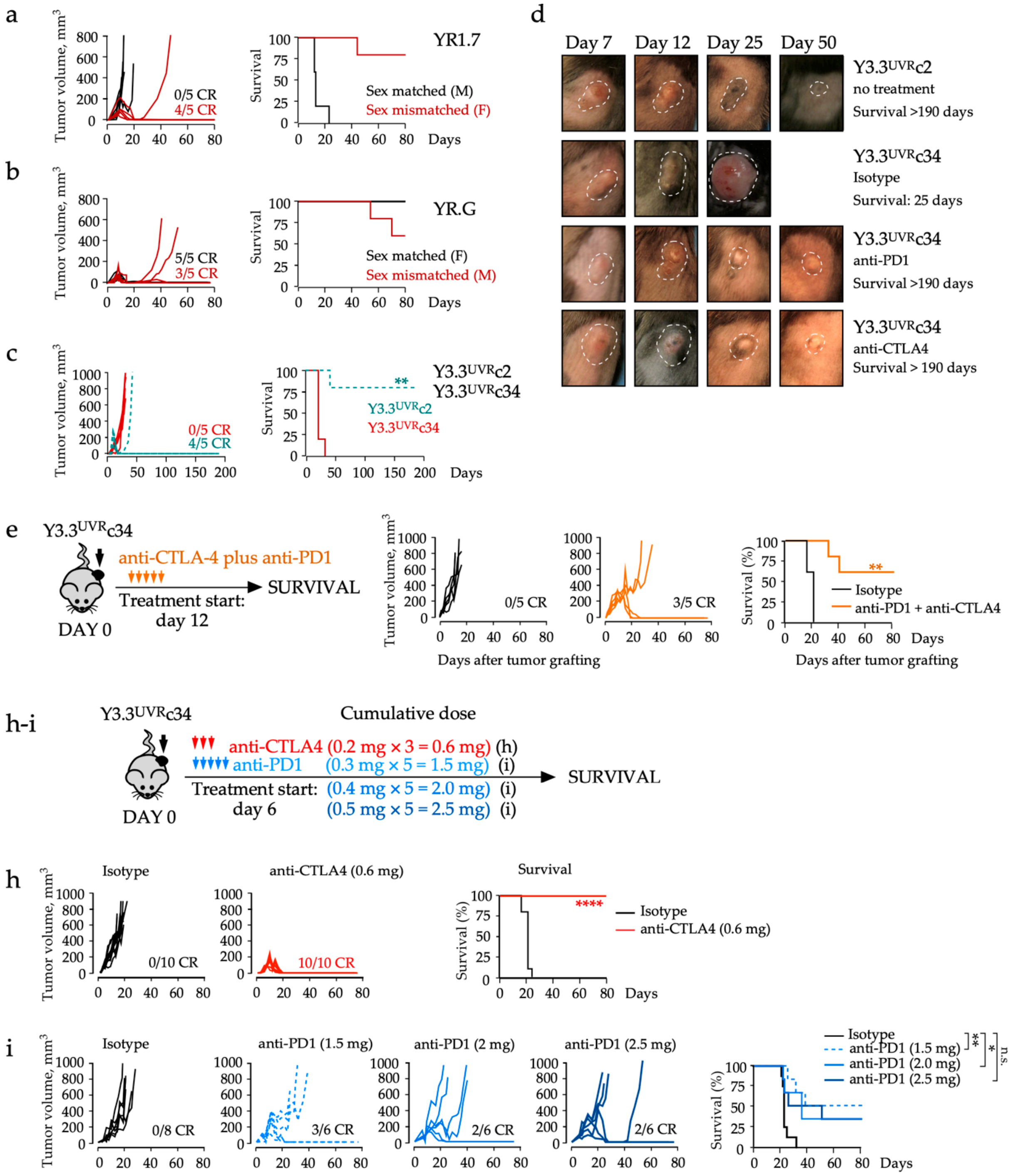

Figure S2. Growth and immunotherapy response patterns of selected YUMM-derived tumors. (**a**,**b**) YR1.7 (a) or YR.G cells (b) were subcutaneously injected into sex-matched or -mismatched C57BL/6 recipients (1x10^6 cells per mouse, n = 5 per group). Tumor growth (left panels) and animal survival (right panels) were monitored. (**c**) Y3.3^{UVR}c2 or Y3.3^{UVR}c34 cells were injected subcutaneously into sex-matched (female) C57BL/6 hosts (0.5x10^6 cells per mouse, n = 5 per group). Tumor growth (left) and animal survival (right); Y3.3^{UVR}c2, teal dashed lines; Y3.3^{UVR}c34, solid red lines. (**d**) Gross appearance Y3.3^{UVR}c2 and Y3.3^{UVR}c34 subcutaneous tumors. Tumors were established and animals

were immunotherapy-treated as described in Figure 2c. Representative longitudinal images are shown. Dashed outlines indicate tumor size. (**e**) Response to combined immunotherapy (anti-PD1 0.3 mg/mouse × 5 plus anti-CTLA4 0.2mg then 0.1mg/mouse × 2) in animals bearing Y3.3^{UVR}c34 tumors; treatment was initiated on day 12. Left to right, experimental set-up; individual tumor growth curves; animal survival (black, isotype control; red, anti-CTLA4; blue, anti-PD1). (**h,i**) Effect of immunotherapy dose escalation on Y3.3^{UVR}c34 tumor growth. Top, experimental set-up: immunotherapy with anti-PD1 (blue) or anti-CTLA4 (red) was initiated on day 6 after tumor cell injection and delivered every 3 days to cumulative dose of 0.6 mg/mouse for anti-CTLA4 (h) and either 1.5 mg, 2 mg or 2.5 mg/mouse for anti-PD1, as indicated (i). Tumor growth (left panels) and animal survival (right panels) are shown. Complete responses (CR) and group sizes are indicated. Data in (c, e, h, i) compared using Mantel-Cox test (*p <0.05, ** p <0.01, **** p <0.0001, n.s. not significant).

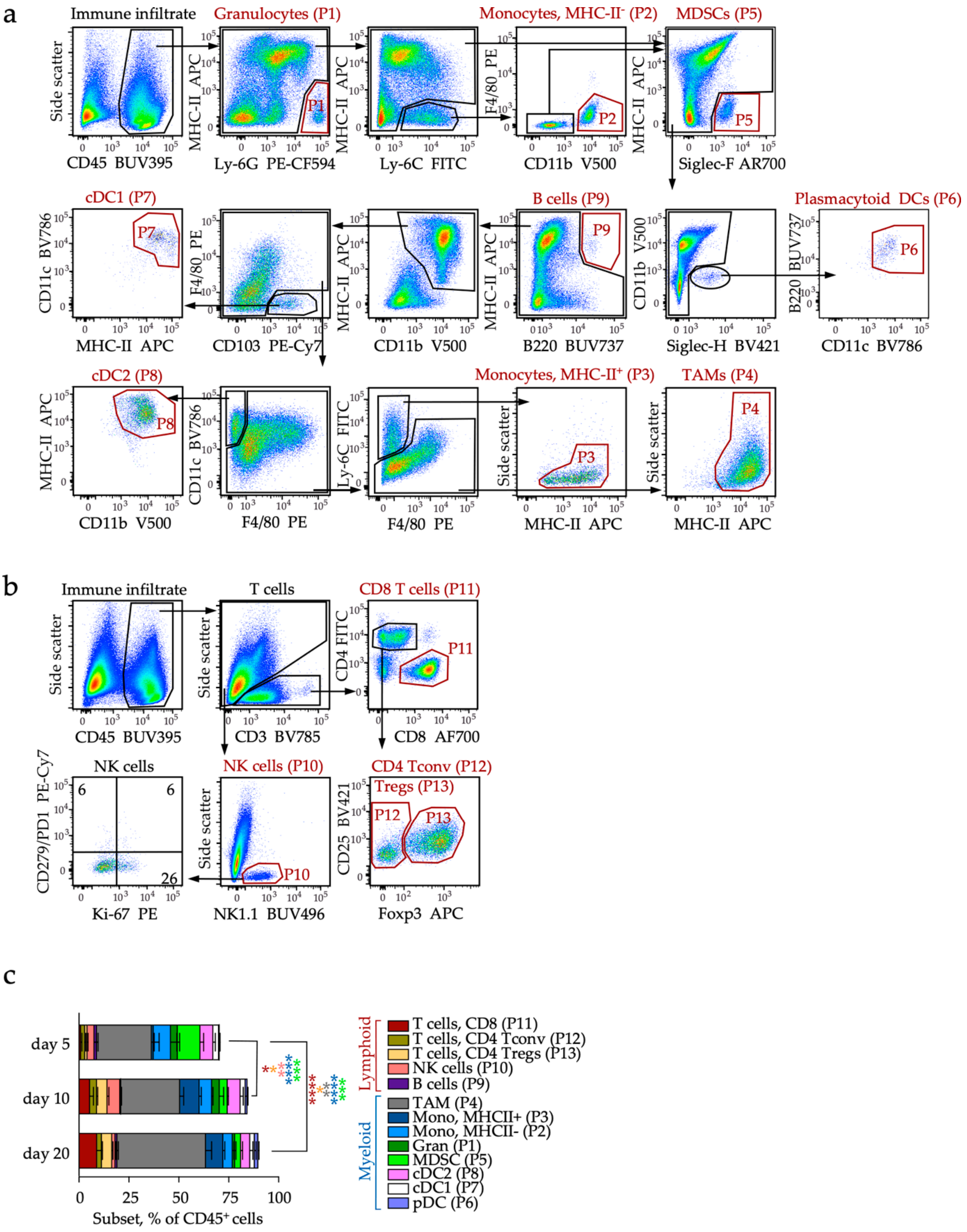

Figure S3. Flow cytometric evaluation of tumor immune contexture. (**a**,**b**) Gating for myeloid (a) and lymphoid (b) subsets in Y3.3^{UVR}c34 tumors. Gates were applied after general gating for time, single cells and viability (not shown). Final gates and the corresponding cell subsets are indicated in red. (**c**) Evolution of Y3.3^{UVR}c34 tumor microenvironment over time (n = 4–6 per group). Data compared using one-way ANOVA with Tukey's multiple comparisons test (*p <0.05; ** p <0.01; *** p <0.001).

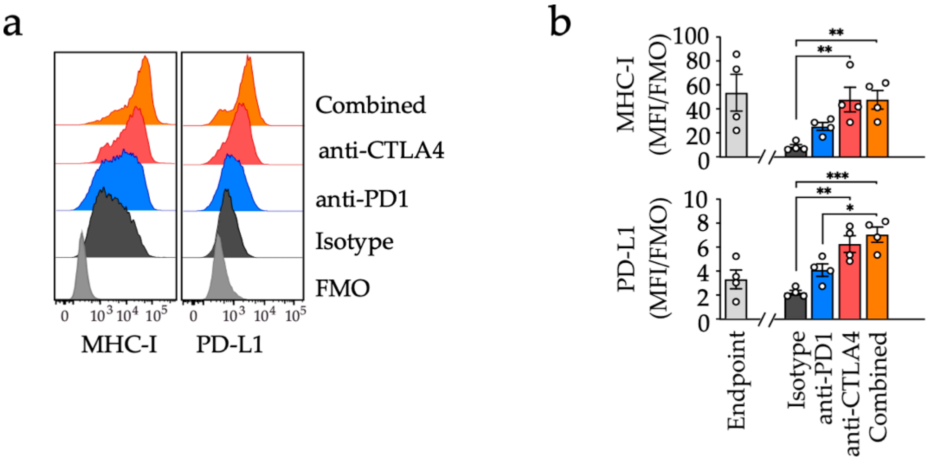

Figure S4. Early on-treatment changes in tumor cell phenotype. In the experiment described in Figure 4, flow cytometric analyses were performed on tumor samples collected after two rounds of immunotherapy. (**a**) Representative histograms and (**b**) expression summary of MHC-I (top) and PD-L1 (bottom) expression on tumor cells. Treatment: isotype control, gray; anti-PD1, blue; anti-CTLA4, red; combination (anti-PD1 plus anti-CTLA4), orange; background (FMO in the respective channel), light gray shaded histograms. Data from large endpoint control tumors from a different experiment are shown for comparison in (b). Data compared using one-way ANOVA with Tukey's multiple comparisons test (**p* <0.05; ** *p* <0.01; *** *p* <0.001).

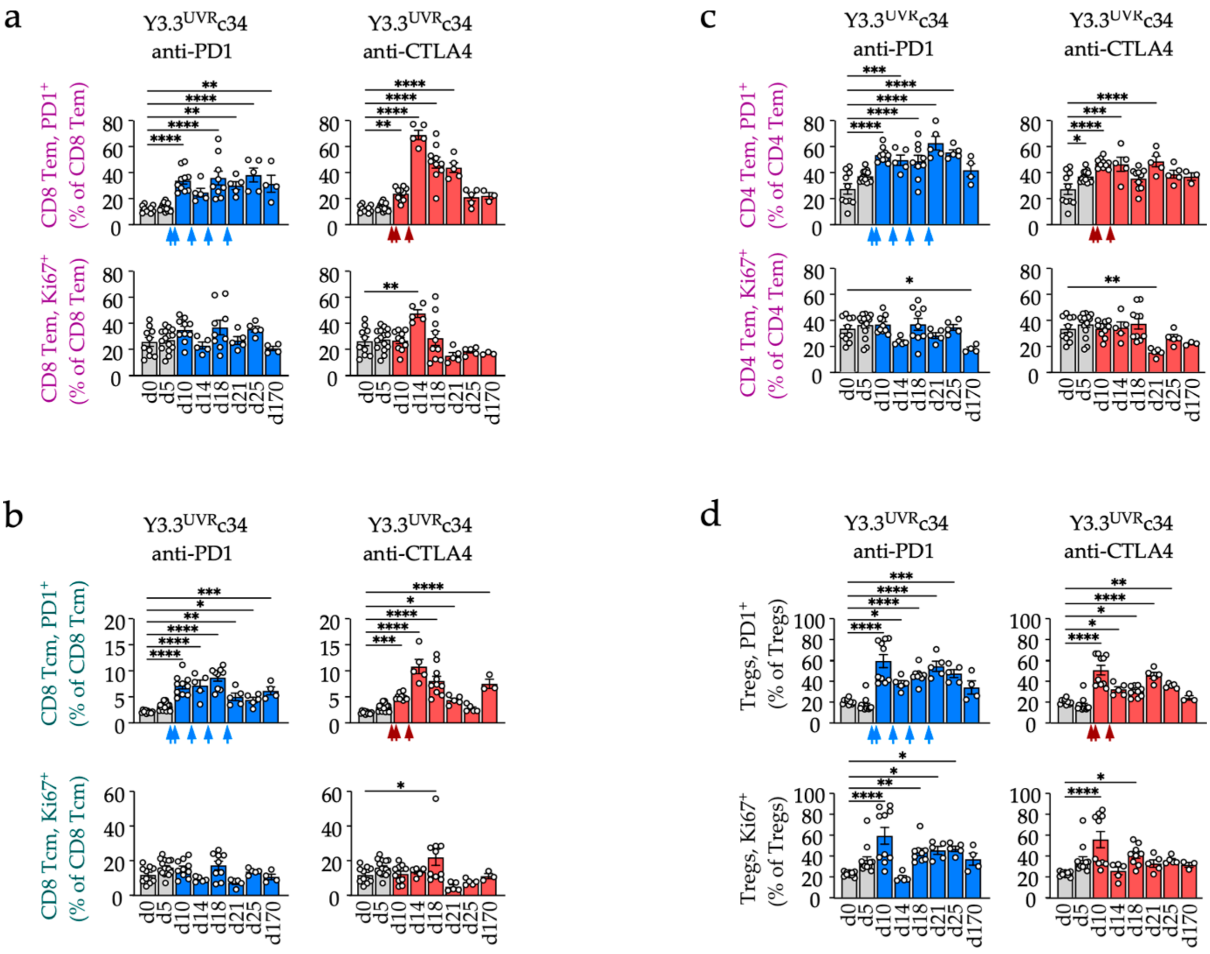

Figure S5. Changes in circulating T-cell phenotypes following immunotherapy with anti-PD1 or anti-CTLA4 antibodies. Animals were grafted with Y3.3^{UVR}c34 tumors and treated as described in Figure 2d. Animals were bled on indicated days, and phenotypes of circulating T cells were assessed by flow cytometry (see Figure 5 for details). (**a**,**b**) PD-1 (top rows) or ki-67 expression (bottom rows) in CD44$^+$CD62L$^-$ CD8 Tem cells (a) and CD44$^+$CD62L$^+$ CD8 Tcm cells (b) in animals treated with

anti-PD1 (blue) or anti-CTLA-4 (red). (**c**) As for (a) but for the CD4 Tem subset. (**d**) As for (a) but for the Treg subset. Data compared using one-way ANOVA with Tukey's multiple comparisons test (**p* <0.05; ** *p* <0.01; *** *p* < 0.001).

Supplementary Table S1. Antibodies and reagents for flow cytometry.

Marker	Fluorescence	Company	Cat#	RRID*	Dilution
mCD3**	BV785	Biolegend	100232	RRID:AB_2562554	1/200
mCD4	FITC	BD	553047	RRID:AB_394583	1/500
mCD8a	AF700	Biolegend	100730	RRID:AB_493703	1/200
mCD11b	V500	BD	562127	RRID:AB_10893815	1/500
mCD11c	BV786	BD	563735	RRID:AB_2738394	1/100
mCD25	BV421	BD	564370	RRID:AB_2738772	1/50
mCD44	V500	BD	560780	RRID:AB_1937316	1/200
mCD45	BUV395	BD	564279	RRID:AB_2651134	1/200
mB220	PE-CF594	BD	562290	RRID:AB_11151901	1/500
mB220	BUV737	BD	612838	RRID:AB_2870160	1/400
mCD62L	BUV737	BD	612833	RRID:AB_2870155	1/250
mCD64	PE	BD	558455	RRID:AB_647241	1/300
mCD103	PE-Cy7	Biolegend	121426	RRID:AB_2563691	1/200
mCD273	PE	BD	557796	RRID:AB_396874	1/200
mCD274	BV421	Biolegend	124315	RRID:AB_10897097	1/150
mCD279	PE-Cy7	Biolegend	135215	RRID:AB_10696422	1/200
mF4/80	PE	Miltenyi	130-116-499	RRID:AB_2727574	1/200
mFc block		BD	553142	RRID:AB_394657	1/100
mFoxp3	APC	TFS	77-5775-40	RRID:AB_469981	1/200
mKi-67	PE	TFS	12-5698-82	RRID:AB_11150954	1/200
LiveDead fixable	Near-InfraRed	TFS	L34976	RRID:SCR_004098	1/100
mLy-6C	FITC	Miltenyi	130-111-777	RRID:AB_2652801	1/500
mLy-6G	PE-CF594	BD	562700	RRID:AB_2737730	1/250
mMHC-I (H-2K^b)	PE	Biolegend	116508	RRID:AB_313735	1/300
mMHC-I (H-2K^b/H-2D^b)	PE	Biolegend	114608	RRID:AB_313599	1/300
mMHC-II (I-A/I-E)	AF647	BD	562367	RRID:AB_11152078	1/250
mMHC-II (I-A/I-E)	AF488	Biolegend	107616	RRID:AB_493523	1/500
mNK1.1	BUV496	BD	741062	RRID:AB_2870674	1/200
mSiglec-F	AR-700	BD	565183	RRID:AB_2739097	1/100
mSiglec-H	BV421	BD	566581	RRID:AB_2739747	1/250
anti-human IgG4-Fc	PE	Southern Biotech	9200-09		1/100
hCD274	BV421	Biolegend	329714	RRID:AB_2563852	1/50
hMHC-I (HLA-A, B, C)	AF700	Biolegend	311438	RRID:AB_2566306	1/80
hKI67	APC	TFS	17-5699-42	RRID:AB 2573218	1/200
hCD274/PD-1 (Pembrolizumab)		Merck			20ug/ml
hFc block		BD	564220	RRID:AB 2869554	1/200

*RRID, Research Resource Identifier; **Specificity: m, mouse; h, human
